# Impact of chronic Achilles tendinopathy on health-related quality of life, work performance, healthcare utilisation and costs

**DOI:** 10.1136/bmjsem-2020-001023

**Published:** 2021-03-26

**Authors:** Tjerk S O Sleeswijk Visser, Arco C van der Vlist, Robert F van Oosterom, Peter van Veldhoven, Jan A N Verhaar, Robert-Jan de Vos

**Affiliations:** 1 Department of Orthopedic Surgery and Sports Medicine, Erasmus MC University Medical Centre, Rotterdam, Netherlands; 2 Department of Orthopedic Surgery, Leiden University Medical Centre, Leiden, Netherlands; 3 Department of Sports Medicine, Haaglanden Medical Centre, Leidschendam, The Netherlands

**Keywords:** tendinosis, economics, sociology, quality of life, Achilles

## Abstract

**Objectives:**

To evaluate the impact of Achilles tendinopathy (AT) on quality of life (QoL), work performance, healthcare utilisation and costs in adults with conservatively treated chronic midportion AT.

**Methods:**

This cross-sectional survey-based study included 80 patients and took place in a sports medicine department of a large regional hospital in the Netherlands. Data were collected before any intervention was given. Primary outcome was the EuroQol questionnaire (EQ-5D). The EQ-5D expresses the percentage of moderate/major problems on the domains self-care, anxiety/depression, mobility, usual activities and pain/discomfort. Secondary outcomes were the number of previous healthcare visits, work performance during the period of symptoms and estimated annual direct medical and indirect costs per patient as a result of AT.

**Results:**

All 80 patients completed the questionnaires. The EQ-5D scores were low for the domains self-care (1%) and anxiety/depression (20%), and high for the domains mobility (66%), usual activities (50%) and pain/discomfort (89%). Patients with AT mainly reported an impact on work productivity (38%). Work absenteeism due to AT was present in 9%. The total median (IQR) number of annual healthcare visits was 9 (3–11). The total mean (SD) estimated annual costs were €840 (1420) per patient with AT (mean (SD) US$991 (1675)).

**Conclusions:**

This study shows the large impact of AT on QoL and work productivity. This study also provides new information about the socioeconomic impact of AT, which emphasises that this common and longstanding disease causes substantial costs. These findings stress the need for optimised treatment and improved preventive interventions for AT.

**Trial registration number:**

NCT02996409.

**What are the new findings?**

- The impact of Achilles tendinopathy (AT) on quality of life is substantial.
- Especially the domains mobility, pain/discomfort and usual activities are affected.
- AT also leads to a significant decrease in work productivity and causes substantial costs.

## Introduction

The term Achilles tendinopathy (AT) entails the clinical triad of localised Achilles tendon pain, tendon thickening and impaired load-bearing capacity.^1–4^ AT is frequently observed in middle-aged, physically active people.^1^ The incidence rate of AT is 2–3 per 1000 Dutch general practice registered patients and has risen in the past decade, probably as a result of an increasing amount of people performing sports activities.^2 5^ Various treatment options are available and conservative treatment is the primary treatment of choice, but is not very effective.^6^ Despite treatment, two-thirds of the patients with new-onset AT remain symptomatic at 1-year follow-up.^7^ At 10-year follow-up, still a quarter of the patients remain symptomatic.^7 8^

The restricting pain and impaired load-bearing capacity associated with AT is assumed to decrease quality of life (QoL).^1 4 9^ Indeed, recent qualitative studies showed that some patients with AT describe profound impact on their life (eg, their identity, social activities and perceived levels of fitness).^10–12^ One of these exploratory studies showed that AT is associated with a lower QoL score compared with normative data.^11^ In this study, however, patients were included online without verifying the diagnosis of AT and the QoL scores were not compared with other musculoskeletal diseases. Additionally, a significant number of patients did not have AT at the time of inclusion, but experienced symptoms suggestive for AT in the past. This could have resulted in recall bias. Other musculoskeletal conditions also affect QoL,^13–16^ with the magnitude of this impact varying among the conditions.^13–18^ It is important to be informed about the magnitude of the impact on QoL of specific diseases to be aware of the urgency on scientific agendas and it also aids in designing management plans when there is knowledge of the specific domains affected. This information is unknown in AT. Knowledge of the impact of AT on work performance, healthcare utilisation and costs is currently also lacking.

The primary aim of this study is to evaluate the impact on QoL in conservatively treated patients with chronic midportion AT. The secondary aims are to assess the effect of AT on (1) work performance, (2) healthcare utilisation and (3) estimated direct and indirect costs. We hypothesised the impact and socioeconomic consequences of AT on QoL to be similar to other musculoskeletal conditions (such as lateral epicondylar tendinopathy, knee osteoarthritis, rheumatoid arthritis and chronic back pain).

## Methods

### Study design

The study was designed at the Erasmus MC University Medical Centre (Rotterdam, the Netherlands) in collaboration with Haaglanden Medical Centre (Leidschendam, the Netherlands). This cross-sectional study was part of a clinical trial, in which this part was completed before any intervention was given. The trial was registered before commencement (ClinicalTrials.gov; NCT02996409).

### Patient and public involvement

Patients or the public were not involved in the design and conduct of the study, the choice of outcome measures or the development of the research questions.

### Patients

The study was conducted at the sports medicine department of a large regional hospital (Haaglanden Medical Centre), from December 2016 to January 2019. A study announcement was made through informing healthcare professionals (both medical and paramedical) and patients via letters, conferences and social media platforms. If patients passed a telephone and online screening, an appointment with the sports medicine physician was planned to assess eligibility. The main inclusion criteria were as follows: (1) age 18–70 years, (2) painful swelling of the midportion of the Achilles tendon (2–7 cm proximal of the calcaneal insertion), (3) symptom duration of more than 2 months and (4) no response to at least 6 weeks of exercise therapy. The main exclusion criteria were an Achilles tendon rupture, clinical suspicion of other tendinopathies (including insertional AT), inability to perform exercise therapy and previous surgical intervention for this condition. The full list of inclusion and exclusion criteria is displayed on ClinicalTrials.gov. Written informed consent was obtained from all subjects before inclusion.

### Procedures

We obtained the outcome measures of this cross-sectional study before any intervention was given. Patients filled in several questionnaires directly following the inclusion appointment with the sports medicine physician. For the clinical trial, patients received either a peritendinous high-volume injection or a placebo injection. The results of this clinical trial have been published elsewhere.^19^

### Outcome measures

#### Primary outcome measure

QoL was measured using the validated Dutch version of the EuroQol questionnaire (EQ-5D-3L).^20^ The EQ-5D-3L consists of five questions involving the following dimensions: mobility, self-care, daily activities, pain and anxiety/mood. Each domain consists of three response options: no problems, moderate problems and major problems. The results of the EQ-5D are dichotomised and expressed as the percentage of subjects with moderate or major problems (any problem).^21^ The EuroQol Visual Analogue Scale (EQ-VAS) was used to evaluate self-rated current overall health status. The EQ-VAS consists of a tape ruler from 0 to 100 (with 0 points being the worst imaginable health status).

#### Secondary outcome measures

We assessed work performance with a questionnaire by asking the number of lost days of work and a decrease in work productivity (yes/no) since the onset of symptoms. We corrected this secondary outcome measure for symptom duration, thereby displaying work performance outcome measures on an annual basis.

Healthcare utilisation was expressed in the total annual number of healthcare visits, the type of healthcare provider and the type of treatment. Participants who reported visiting a healthcare provider, but could not specify the number of visits or treatments, were recorded as missing data. Participants who reported visiting a sports medicine physician or orthopaedic surgeon were assumed to have at least one consultation with a general practitioner (GP), as a referral from a GP to a medical specialist (eg, sports physician or orthopaedic surgeon) is required in the Netherlands. Participants who reported treatment with a certain number of injections, but did not specify the number of visits to a medical specialist, were assumed to have an equal number of visits to a medical specialist as the number of injections.

We divided costs into two categories: direct costs as a result of medical consumption and indirect costs as a result of lost working days or decreased work productivity. The direct medical costs were calculated with the following formula: total number of visits/treatments multiplied with estimated medical costs for those visits/treatments. In 2016, the Dutch Healthcare Authority published a guideline for economic evaluations in healthcare.^22^ Using this guideline, we established medical costs per visit/treatment and estimated productivity costs per hour at €34.75 (US$38.57) per person.^22 23^ Costs used for the economic evaluation are specified in online supplemental file 1. Costs in dollars were calculated using the average exchange rate of the respective study period. We did not register the profession of the patients and therefore did not adjust the costs for type of profession. Indirect costs were calculated by lost working days/work productivity multiplied by the costs per working day. Costs per working day were calculated using the productivity costs per hour. To calculate indirect costs due to a decrease in work productivity, we estimated reduced productivity without sickness absence at 1.0 hour per month. This is based on previous research on self-reported productivity loss in patients with musculoskeletal disorders.^24^ The annual direct and indirect costs were adjusted for symptom duration because we asked patients about these costs during their symptomatic period.

10.1136/bmjsem-2020-001023.supp1 Supplementary data

### Statistical analysis

We assessed data for having a normal distribution using the Shapiro-Wilk test. Normally distributed data are presented as mean with SD and non-normally distributed data as median with IQR. We chose to present costs (both in € and US$) as mean with SD, as we wanted to include the weight of outliers on both sides. A median value is also presented to provide a better interpretation of these data and improve the comparability to other studies. Completeness of data is specified in online supplemental file 2. We used SPSS software (V.24.0.0.1; SPSS) for statistical analysis. For the randomised controlled trial, of which this study was part, we performed a sample size calculation based on the primary outcome of the study. We estimated that 80 patients were needed to detect a clinically relevant between-group difference in the primary outcome.^19^ As post hoc power analyses are discouraged and this is a descriptive study, we refrained from performing an additional power calculation.

10.1136/bmjsem-2020-001023.supp2 Supplementary data

## Results

### Patient population

All 80 patients that were included in the clinical trial completed the questionnaires for this cross-sectional study (missing data 0%). The median (IQR) age in our study population was 50 (44–54) years, with 39 participants being male (49%). The median (IQR) body mass index (BMI) was 25.7 kg/m^2^ (23.9–30.0) and median (IQR) symptom duration was 63 weeks (40–127). All registered patient characteristics are shown in table 1.

**Table 1:** Descriptive statistics of participants

Characteristics (n=80)	Mean (SD)/Median (IQR)
**Personal characteristics**	
Age (years)	50 (44–54)
Sex (male/female)	39/41
BMI (kg/m^2^)	25.7 (23.9–30.0)
**Injury-related factors**	
AT (unilateral/bilateral; n)	52/28
Symptom duration (weeks)	63 (40–127)
VISA-A score (0–100)	42.8 (15.8)
**Sports-related factors**	
Sports duration (hours/week)	4 (2.5–6.0)
AAS score (0–10)	5 (5.0–6.0)
Sport adaptation (none/reduced/stopped; n)	2/22/56
**Work-related factors**	
Sedentary work per working day (%)	68 (36–80)

Values are displayed in frequencies and medians (IQR)/means (SD).

Sports adaptation: patients who reported no change in sports activities, a reduce of sports activities or stopped performing sports activities.

VISA-A: A score range from 0 to 100 points (with asymptomatic persons expected to score 100 points) used for assessment of physical disability due to AT.^39^

AAS: A score range from 0 to 10 points (with 0 being unable to walk and 10 being physically active performing high-intensity sports on a top level) which includes different sports, working activities and general activities used to assess the level of activity in persons.^40^

AAS, Ankle Activity Score; AT, Achilles tendinopathy; BMI, body mass index; VISA-A, Victorian Institute of Sport Assessment Achilles.

### Primary outcome: QoL

The majority of patients with AT reported moderate or major problems (any problem) on the domains mobility (66%), usual activities (50%) and pain/discomfort (89%). Low frequencies were reported for the domains self-care (1%) and depression/anxiety (20%). Table 2 shows the distribution of EQ-5D scores in patients with AT. Median (IQR) self-rated current overall health-status using the EQ-VAS score was 70 points (59–80).

**Table 2:** EQ-5D scores in patients with Achilles tendinopathy

N=80	No problems	Moderate problems	Severe problems
Mobility	27 (34%)	52 (65%)	1 (1%)
Self-Care	79 (99%)	1 (1%)	0 (0%)
Usual activities	40 (50%)	39 (49%)	1 (1%)
Pain/discomfort	9 (11%)	63 (79%)	8 (10%)
Anxiety/depression	64 (80%)	14 (18%)	2 (2%)

Displayed values are the number of patients (%).

EQ-5D, EuroQol questionnaire.

### Secondary outcomes

#### Work performance

Work absenteeism due to AT was reported in 9% of the patients. Within this 9%, the mean (SD) annual number of days that patients were unable to work due to AT was 7.8 (5.7). The median (IQR) annual number of days that the whole study population of patients was unable to work due to AT was 0 (0–0). Thirty-eight per cent of the patients reported a decrease in work productivity.

#### Healthcare utilisation

The median (IQR) total number of healthcare visits was 9^3–11^ per patient per year. The majority (84%) reported having visited a physiotherapist and 23% reported the use of foot orthoses prescribed by a podiatrist. Thirty-nine per cent visited a GP, whereas 28% of the participants visited a sports medicine physician or orthopaedic surgeon. Thirty-three per cent of all annual healthcare visits consisted of ‘regular physiotherapy treatment’ (eg, exercise therapy, massage therapy and taping) performed by a physiotherapist. Table 3 demonstrates the frequencies of annual healthcare visits per type of healthcare provider. Table 4 shows the annual healthcare utilisation per type of treatment.

**Table 3:** Annual healthcare utilisation and medical costs per patient, per type of healthcare provider (n=80)

Healthcare provider	Patients using resource, n (%)	Mean resource consumption (% of all healthcare visits)	Mean (SD) medical costs	Median (IQR) medical costs
**Primary care (visits**)				
General practitioner	31 (39)	0.50 (4.6)	€17 (47)	€0 (0–17)
Physical therapist	67 (84)	9.7 (88.2)	€320 (598)	€176 (33–355)
Podiatrist	18 (23)	0.15 (1.4)	€23 (51)	€0 (0–0)
Other*	6 (8)	0.27 (2.4)	€20 (94)	€0 (0–0)
**Secondary care (visits**)				
Sports medicine physician/orthopaedic surgeon	22 (28)	0.37 (3.4)	€36 (71)	€0 (0–42)
**Total**		10.8 (100)†	€415 (631)	€258 (131–480)

Differences between healthcare visits/costs and total visits/costs are due to rounding off.

*Another healthcare provider (eg, osteopath, chiropractor or complementary medicine).

†Total median (IQR) annual healthcare visits was 9 (3–11).

**Table 4:** Annual healthcare utilisation and medical costs per patient, per type of treatment (n=80)

Healthcare resource	Patients using resource, n (%)	Mean resource consumption (% of all healthcare visits)	Mean (SD) medical costs	Median (IQR) medical costs
**Treatments (units**)				
Physiotherapy*	67 (84)	3.6 (33)	€120 (363)	€0 (0–156)
Shockwave	35 (44)	2.6 (24)	€86 (222)	€0 (0–99)
Acupuncture/dry needling	16 (20)	1.7 (16)	€55 (267)	€0 (0–0)
Laser therapy/EPTE	7 (9)	0.33 (3)	€11 (47)	€0 (0–0)
Injection therapy†	8 (10)	0.06 (0.6)	€2 (9)	€0 (0–0)

*‘Regular physiotherapy treatment’ (eg, exercise therapy, massage therapy and taping) performed by a physiotherapist.

†Prolotherapy, platelet-rich plasma or corticosteroids.

EPTE, therapeutic percutaneous electrolysis.

#### Estimated direct and indirect costs

The mean (SD) total healthcare costs were €415 (631) (US$490) per patient per year (median (IQR) €258 (131–480)). Physiotherapy treatments accounted for 77% of the total healthcare costs. Annual costs for healthcare use per type of healthcare provider are presented in table 3. The annual costs per type of treatment are demonstrated in table 4. Costs in US dollars (US$) are specified in online supplemental tables 3 and 4.

10.1136/bmjsem-2020-001023.supp3 Supplementary data

10.1136/bmjsem-2020-001023.supp4 Supplementary data

In patients who reported a decrease in work productivity, annual costs due to reduced work productivity were €417 (US$463) per employee. Total mean (SD) costs due to absenteeism and productivity loss are €425 (1319) (US$501) per patient with AT per year. Total mean (SD) estimated annual direct and indirect costs are €840 (1420) (US$991) per patient with AT. Costs from loss of work productivity and absenteeism accounted for 51% of the total costs.

## Discussion

We demonstrated in this cross-sectional study that AT is associated with a low QoL score, specifically on the domains mobility, usual activities and pain/discomfort. Work absenteeism due to AT was low (reported in 9% of the patients), whereas more than one-third of the patients (38%) reported a reduction in work productivity due to AT. The total median annual number of healthcare visits was nine and the total mean estimated annual direct and indirect costs are €840 (US$991) per patient with AT.

### Quality of life

**Figure 1:**
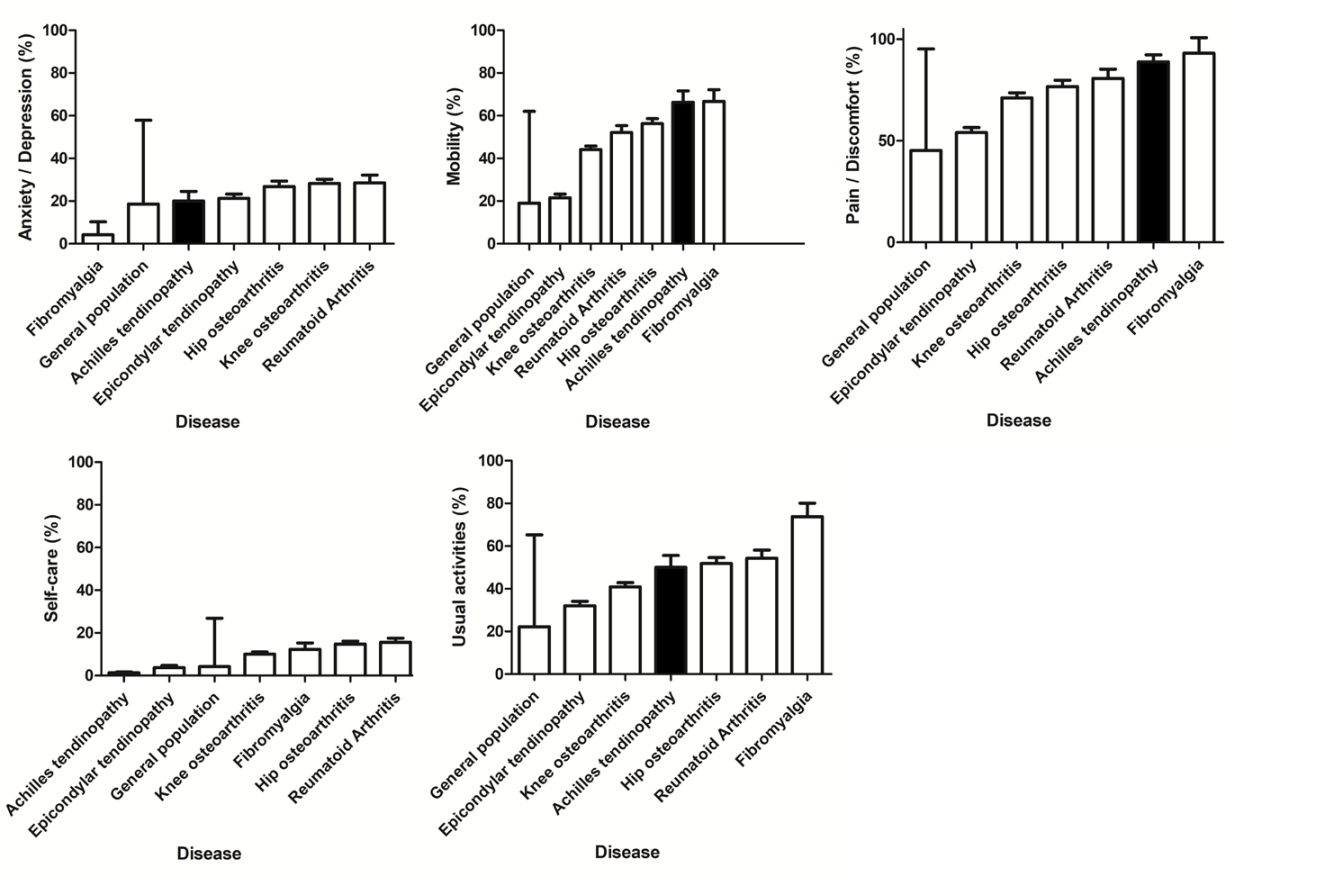
The EQ-5D scores for persons with musculoskeletal diseases per domain. DMC_3_ study.^16^ Displayed values are percent with any (moderate and severe) problems (SE). EQ-5D: EuroQoL five-item questionnaire for measuring health-related quality of life. General population: EQ-5D score in a large sample of the general population (no target on specific diseases) aged ≥25 years (n=3664), weighted for age and sex in the Dutch population of 1998. DMC_3_ study, Dutch population-based musculoskeletal complaints and consequences cohort study.

The finding of a low QoL score in patients with AT is in line with a recent exploratory study.^11^ The median EQ-VAS score in the current study was comparable to patients with chronic patellar tendinopathy (70 points vs 68 points).^25^ We also compared the score from the health-related QoL measure (EQ-5D) for AT to a large sample (n=3664) of the general Dutch population and different musculoskeletal diseases.^16^ Having AT was associated with a worse mean QoL score, compared with those without a musculoskeletal disease, on all EQ-5D dimensions, except for self-care. Patients with AT reported a similar, if not worse, QoL score on the domains mobility, usual activities and pain/discomfort, compared with those with other musculoskeletal diseases, such as rheumatoid arthritis, osteoarthritis, lateral epicondylar tendinopathy (tennis elbow) and fibromyalgia. Figure 1 depicts the differences in QoL domain scores between these diseases.^16^

### Impact on work

Rotator cuff tendinopathy, lateral epicondylar tendinopathy and patellar tendinopathy all negatively impact work productivity and result in increased rates of absence from work.^26–30^ Work performance in patients with AT was frequently decreased because of reduced work productivity. This is similar to research on upper extremity musculoskeletal disorders.^31^ In patients with lateral epicondylar tendinopathy and rotator cuff tendinopathy, 56% had decreased work productivity, while decrease in work productivity due to AT in our study was lower with 38%.^29 32^ The impact of AT on work productivity is comparable to moderate knee osteoarthritis and patellar tendinopathy (40% and 36% decreased work productivity, respectively).^29^ The majority of the patient population in our study performed sedentary work (68%), which is conceivably less impacted by AT. The impact of AT may thus even be higher in populations with physical work.

### Healthcare utilisation and costs

Healthcare utilisation is an important measure for public healthcare organisations. The burden is especially large in individuals with chronic pain conditions.^33^ One previous study examined the healthcare utilisation and costs for patients with lateral epicondylar tendinopathy.^34^ Median number of annual physiotherapy visits was higher in patients with AT compared with patients with lateral epicondylar tendinopathy (seven for AT vs three for lateral epicondylar tendinopathy), while the median number of medical specialist visits was comparable (one for both disorders).^34^

Both indirect costs due to the inability to work and direct costs as a result of tendinopathy have not been extensively researched. In the USA, direct semiannual medical costs for conservatively treated patients with lateral epicondylar tendinopathy were US$168 (€151) per patient.^34^ The total median annual medical costs per patient were slightly higher in conservatively treated patients with lateral epicondylar tendinopathy, knee osteoarthritis and ankylosing spondylitis compared with conservatively treated patients with AT (€305, €660 and €451 vs €258, respectively).^34–36^ Patients with fibromyalgia and chronic back pain reported slightly lower median annual medical costs for primary and secondary care compared with patients with AT (€190 and €131 vs €258, respectively).^36^

Socioeconomic consequences of patients with AT for the public are substantial, based on Dutch incidence rates of AT and the persisting nature of the condition.^7 8^ The absolute socioeconomic burden of AT in the Netherlands can be estimated at more than 21 million euros. Based on an incidence rate of 2.35 per 1000 in general practice registered adult patients and a total of 5028 general practices (with an average of 2095 patients per practice) in the Netherlands, the total number of annual new Dutch AT patients is estimated at 25 000.^5^ The total socioeconomic burden can therefore be estimated at 25 000×€840 = €21 000 000 (US $24 780 000). This is likely to be an under-representation, as our study shows that only 39% of these patients visit a GP and it is known that in an open population of runners sustaining AT, the majority is seeking other sources of primary healthcare than general practice (eg, physiotherapy).^37^

Previous research indicated that surgery is performed in up to 24% of all patients with AT in some countries.^38^ Surgically treated patients with AT were excluded in our study. Including these would lead to a significant increase in healthcare costs. Furthermore, we did not use costs of medication use and imaging in the comparison as we, contrary to the other studies, did not collect this information. An illustration of the possible impact if imaging costs were included in this study is provided in online supplemental file 3. It is conceivable that work absence, healthcare utilisation and healthcare costs would also be significantly higher if surgically treated patients were included and medication use and imaging costs would have been included. Therefore, the actual impact of AT on work performance, healthcare utilisation, and direct and indirect costs may be even larger than presented in this study.

### Strengths and limitations

Our study is one of the first studies to evaluate the impact of AT on QoL, work performance, healthcare utilisation and estimated direct and indirect costs. To assess the impact of AT on QoL, we used the reliable and validated EQ-5D questionnaire.^20^ Data were complete for our primary outcome and were retrieved from a homogeneous group of clinically diagnosed patients with AT. However, there are some limitations to this study. We asked patients about the duration of their symptoms and applied treatments retrospectively, which could have induced recall bias on these specific items. This may have resulted in inaccuracy in collection of these secondary outcome measures.

Second, loss in work productivity was measured using a binary response option (‘yes’ or ‘no’), the amount of loss of work productivity was not specified. Third, the study population may not be representative of all patients with AT. Most patients included in this study had longstanding symptoms (median 63 weeks) and it is likely that patients with short living AT experience less impact on QoL, work performance and visit less healthcare providers. Another limitation was that the direct costs were mainly based on assumptions of the national mean costs of treatments. The main reason for this is that we did not register accurate data of the profession of the patients. This might have provided a less accurate estimation of the direct costs.

### Recommendations for future research

Our main recommendation for future research is to evaluate the effect of different treatments on QoL scores in patients with AT. This will gain more insight into the impact and effectiveness of different treatments. Second, it would be interesting to investigate the cost-effectiveness of different treatments. To better understand the economic impact of AT, future studies could research the specific underlying cause of the decreased work productivity.

## Conclusion

We demonstrated the large impact of AT on QoL, specifically on the domains mobility, pain/discomfort and usual activities. The magnitude of this impact seems similar to other chronic musculoskeletal conditions, such as knee osteoarthritis and rheumatoid arthritis. AT impacts significantly on work, with more than one-third of patients having decreased work productivity. Healthcare utilisation, direct and indirect costs as a result of AT are substantial with a total mean estimated annual direct and indirect costs of €840 per patient with AT. These costs seem similar to other chronic musculoskeletal conditions. The above-mentioned socioeconomic impact of AT stresses the need for optimised treatment and improved preventive measures.
