# Supplementary material for: Impact of chronic Achilles tendinopathy on health-related quality of life, work performance, healthcare utilisation and costs
Source: BMJ Open Sport Exerc Med. 2021 Mar 26;7(1):e001023. doi: 10.1136/bmjsem-2020-001023 (PMC8006822; doi:10.1136/bmjsem-2020-001023)
Supplement: Supplementary data [file bmjsem-2020-001023supp001.pdf]

Supplementary file 1. Cost used in the economic evaluation

Article title: Impact of chronic Achilles tendinopathy on health-related quality of life, work performance, healthcare utilization, and costs

Journal name: BMJ Open Sport & Exercise Medicine

Authors: Tjerk SO Sleeswijk Visser, Arco C van der Vlist, Robert F van Oosterom, Peter LJ van Veldhoven, Jan AN Verhaar, Robert-Jan de Vos

Affiliation and e-mail address of the corresponding author: Department of Orthopedics and Sports Medicine, Erasmus MC University Medical Centre, email: t.sleeswijkvisser@erasmusmc.nl

Costs used in the economic evaluation are presented in Table 1. We established medical costs per visit to a General Practitioner, sports physician/orthopedic surgeon or physiotherapist based on a guideline for economic evaluations in healthcare published by the Dutch Healthcare Authority.(1, 2) In 2018 shockwave therapy was gathered under regular physiotherapy.(3) To prevent overestimating we gathered Dry Needling and Laser therapy under regular therapy as well. Healthcare costs per visit to a podiatrist were estimated using an independent healthcare insurance comparator. (4)

Direct healthcare costs per visit	Euro's
General practitioner (<20 min)	33.0 ($37)
Sports Physician/Orthopedic Surgeon)	91.0 ($101)
Physiotherapist (intake)	33.0 ($37)
Shockwave therapy	33.0 ($37)
Laser therapy or EPTE	33.0 ($37)
Dry Needling	33.0 ($37)
Injection	40.06 ($44)
Podiatrist	150.0 ($167)

Table 1. Costs used in the economic evaluation

References

1. IJzerman MJ ea. Richtlijn voor het uitvoeren van economische evaluaties in de gezondheidszorg. Door Zorginstituut Nederland. In samenwerking met het ministerie van VWS. . 2016.
2. Hakkaart-van Roijen L VdLN, Bouwmans C, Kanters T, Tan SS. . Kostenhandleiding.

Methodologie van kostenonderzoek en referentieprijzen voor economische evaluaties in de gezondheidszorg. In opdracht van Zorginstituut Nederland. Geactualiseerde versie 2015.
3. Factsheet De rekening van de fysiotherapeut, p.2. https://puc.overheid.nl/nza/doc/PUC_3372_22/1/ Accessed on 13-04-2020.
4. https://www.independer.nl/zorgverzekering/vergoedingen/steunzolen.aspx Accessed on 13-04-2020.
