# Supplementary material for: Impact of chronic Achilles tendinopathy on health-related quality of life, work performance, healthcare utilisation and costs
Source: BMJ Open Sport Exerc Med. 2021 Mar 26;7(1):e001023. doi: 10.1136/bmjsem-2020-001023 (PMC8006822; doi:10.1136/bmjsem-2020-001023)
Supplement: Supplementary data [file bmjsem-2020-001023supp002.pdf]

Supplementary file 2 Completeness of data

Article title: Impact of chronic Achilles tendinopathy on health-related quality of life, work performance, healthcare utilization, and costs

Journal name: BMJ Open Sport & Exercise Medicine

Authors: Tjerk SO Sleeswijk Visser, Arco C van der Vlist, Robert F van Oosterom, Peter LJ van Veldhoven, Jan AN Verhaar, Robert-Jan de Vos

Affiliation and e-mail address of the corresponding author: Department of Orthopedics and Sports Medicine, Erasmus MC University Medical Centre, email: t.sleeswijkvisser@erasmusmc.nl

	**Completeness of data (%)**
**EQ-5D score**	80/80 (100)
**Type of healthcare provider**	
Physiotherapist	47/67 (75%)
Sports physician/Orthopedic surgeon	22/22 (100%)
General Practitioner	31/31 (100%)
Podiatrist	18/18 (100%)
Other	6/6 (100%)
**Type of treatment**	
Physiotherapy	47/67 (75%)
Shockwave	28/35 (80%)
Acupuncture/Dry Needling	12/16 (75%)
Laser therapy/EPTE	6/7 (86%)
Injection	8/8 (100%)

Table 1. Completeness of data for the EQ-5D score and per type of healthcare provider/treatment.
