# Supplementary material for: Impact of chronic Achilles tendinopathy on health-related quality of life, work performance, healthcare utilisation and costs
Source: BMJ Open Sport Exerc Med. 2021 Mar 26;7(1):e001023. doi: 10.1136/bmjsem-2020-001023 (PMC8006822; doi:10.1136/bmjsem-2020-001023)
Supplement: Supplementary data [file bmjsem-2020-001023supp003.pdf]

Supplementary file 3

Article title: Impact of chronic Achilles tendinopathy on health-related quality of life, work performance, healthcare utilization, and costs

Journal name: BMJ Open Sport & Exercise Medicine

Authors: Tjerk SO Sleeswijk Visser, Arco C van der Vlist, Robert F van Oosterom, Peter LJ van Veldhoven, Jan AN Verhaar, Robert-Jan de Vos

Affiliation and e-mail address of the corresponding author: Department of Orthopedics and Sports Medicine, Erasmus MC University Medical Centre, email: t.sleeswijkvisser@erasmusmc.nl

Costs for additional imaging modalities requested by a medical specialist (sports physician/orthopedic surgeon) are presented in Table 1. This table illustrates three different scenario's to gain insight in the possible impact of including imaging costs in our study. In scenario 1 none of the participants had an ultrasound or MRI. In scenario 2 all of the participants had an ultrasound of the lower leg. In scenario 3 all participants had a MRI of the lower leg. Costs for ultrasound (€119,47) and MRI (€424,93) are based on standard costs for those imaging modalities in the Netherlands.

**Secondary Care** (Sports medicine physician/orthopedic surgeon)	**Mean (SD) medical costs**	**Median (IQR) medical costs**
**Scenario 1** (None of the participants received imaging)	€36 (71)	€0 (0-42)
**Scenario 2** (All participants received an ultrasound)	€155 (71)	€119 (119-161)
**Scenario 3** (All participants received a MRI)	€461 (71)	€425 (424-467

Table 1. Additional annual medical costs as a result of imaging studies per patient.
