# Supplementary material for: Impact of chronic Achilles tendinopathy on health-related quality of life, work performance, healthcare utilisation and costs
Source: BMJ Open Sport Exerc Med. 2021 Mar 26;7(1):e001023. doi: 10.1136/bmjsem-2020-001023 (PMC8006822; doi:10.1136/bmjsem-2020-001023)
Supplement: Supplementary data [file bmjsem-2020-001023supp004.pdf]

Supplementary file 4. Costs in dollars ($). Calculated using the average exchange rate of 2018 (1.18)

Article title: Impact of chronic Achilles tendinopathy on health-related quality of life, work performance, healthcare utilization, and costs

Journal name: BMJ Open Sport & Exercise Medicine

Authors: Tjerk SO Sleeswijk Visser, Arco C van der Vlist, Robert F van Oosterom, Peter LJ van Veldhoven, Jan AN Verhaar, Robert-Jan de Vos

Affiliation and e-mail address of the corresponding author: Department of Orthopedics and Sports Medicine, Erasmus MC University Medical Centre, email: t.sleeswijkvisser@erasmusmc.nl

**Health care provider**	**Patients using resource, no. (%)**	**Mean resource consumption (% of all healthcare visits)**	**Mean (SD) medical costs**	**Median (IQR) medical costs**
**Primary care (visits)**				
General Practitioner	31 (39)	0.50 (4.6)	$20 (55)	$0 (0-20)
Physical therapist	67 (84)	9.7 (88.2)	$378 (706)	$208 (39-419)
Podiatrist	18 (23)	0.15 (1.4)	$27 (60)	$0 (0-0)
Other†	6 (8)	0.27 (2.4)	$24 (111)	$0 (0-0)
**Secondary Care (visits)**				
Sports medicine physician/orthopedic surgeon	22 (28)	0.37 (3.4)	$42 (84)	$0 (0-50)
**Total**		10.8 (100)*	$490 (745)	$304 (155-566)

Table 1. Annual health care utilization and medical costs per patient, per type of healthcare provider (n=80)

Abbreviations: IQR: interquartile range, SD: standard deviation

***** Total median (IQR) annual healthcare visits was 9 (3-11).

† Another healthcare provider (e.g. osteopath, chiropractor or alternative medicine).

Differences between healthcare visits/costs and total visits/costs are due to rounding off

**Health care resource**	**Patients using**	**Mean resource**	**Mean (SD)**	**Median (IQR)**

	resource, no. (%)	consumption (% of all healthcare visits)	medical costs	medical costs
**Treatments (units)**				
Physiotherapy*	67 (84)	3.6 (33)	$142 (428)	$0 (0-184)
Shockwave	35 (44)	2.6 (24)	$101 (262)	$0 (0-117)
Acupuncture/dry needling	16 (20)	1.7 (16)	$65 (315)	$0 (0-0)
Laser therapy/EPTE	7 (9)	0.33 (3)	$13 (55)	$0 (0-0)
Injection therapy†	8 (10)	0.06 (0.6)	$2 (11)	$0 (0-0)

Table 2. Annual health care utilization and medical costs per patient, per type of treatment (n=80)
Abbreviations: IQR: interquartile range, SD: standard deviation. EPTE: therapeutic percutaneous electrolysis
*** '**Regular physiotherapy treatment' (e.g. exercise therapy, massage therapy and taping) performed by a physiotherapist.
† Prolotherapy, platelet-rich plasma or corticosteroids
